# Shaping the future of child and adolescent psychiatry

**DOI:** 10.1186/s13034-019-0279-y

**Published:** 2019-04-11

**Authors:** Norbert Skokauskas, Daniel Fung, Lois T. Flaherty, Kai von Klitzing, Dainius Pūras, Chiara Servili, Tarun Dua, Bruno Falissard, Panos Vostanis, María Beatriz Moyano, Inna Feldman, Ciaran Clark, Vlatka Boričević, George Patton, Bennett Leventhal, Anthony Guerrero

**Affiliations:** 1 Word Psychiatric Association, Child and Adolescent Psychiatry Section and Norwegian University of Science and Technology, Trondheim, Norway; 2 The International Association for Child and Adolescent Psychiatry and Allied Professions and Institute of Mental Health, Singapore, Singapore; 3 International Society for Adolescent Psychiatry and Psychology and Harvard University, Cambridge, USA; 4 World Association for Infant Mental Health and University Hospital Leipzig, Leipzig, Germany; 5 Special Rapporteur on the Right of Everyone to the Enjoyment of the Highest Attainable, Standard of Health, United Nations and University of Vilnius, Vilnius, Lithuania; 6 WHO Focal Point for Child and Adolescent Mental Health, Department of Mental Health and Substance Abuse, WHO, Geneva, Switzerland; 7 Department of Mental Health and Substance Abuse, WHO, Geneva, Switzerland; 8 Université Paris-sub, Paris, France; 9 University of Leicester, Leicester, UK; 10 Hospital Francés de Buenos Aires, Buenos Aires, Argentina; 11 Uppsala University, Uppsala, Sweden; 12 Health Service Executive, Dublin, Ireland; 13 Psychiatric Hospital for Children and Youth, Zagreb, Croatia; 14 University of Melbourne, Melbourne, Australia; 15 Department of Psychiatry, University of California San Francisco, San Francisco, USA; 16 University of Hawaii, ‎Honolulu, USA

## Abstract

Child and adolescent psychiatry is in a unique position to respond to the growing public health challenges associated with the large number of mental disorders arising early in life, but some changes may be necessary to meet these challenges. In this context, the future of child and adolescent psychiatry was considered by the Section on Child and Adolescent Psychiatry of the World Psychiatric Association (WPA CAP), the International Association for Child and Adolescent Psychiatry and Allied Professions (IACAPAP), the World Association for Infant Mental Health (WAIMH), the International Society for Adolescent Psychiatry and Psychology (ISAPP), the UN Special Rapporteur on the Right to Health, representatives of the WHO Department of Mental Health and Substance Abuse, and other experts. We take this opportunity to outline four consensus priorities for child and adolescent psychiatry over the next decade: increase the workforce necessary for providing care for children, adolescents and families facing mental disorders; reorienting child and adolescent mental health services to be more responsive to broader public health needs; increasing research and research training while also integrating new research finding promptly and efficiently into clinical practice and research training; Increasing efforts in advocacy.

## Introduction

Children and adolescents constitute about one-third of the world’s population [1]. They are a particularly vulnerable group for the onset of mental disorders [2]. Approximately one-half of all mental disorders emerge before 14 years of age and 75% by 25 years [2, 3]. Furthermore, globally, one-quarter of disability-adjusted life years (DALYs) for mental and substance use disorder occurs in youth [4].

Historically, child and adolescent psychiatry has been the principal medical specialty focused on the mental health of children and adolescents and their families. After a slow emergence in the mid-nineteenth century, child and adolescent psychiatry became a recognized medical specialty early in the twentieth century. It has progressed on many fronts in early years of the last century from differing and opposing views about psychology and philosophy, as well as from empirical discoveries. The recognition of the psychiatric needs of children began with the first child guidance clinic, started by William Healy in 1909. This was sustained by the later establishment of the child psychiatry clinic at Johns Hopkins University and the first textbook on child psychiatry, both by Leo Kanner. In addition, interest in developmental psychopathology was fostered by the development of child psychoanalysis, pioneered by Melanie Klein and Anna Freud, Piaget’s work on cognitive development, Vygotsky’s on psychosocial development and Bowlby’s attachment framework [5–7]. As it developed, child and adolescent psychiatry integrated elements from many disciplines, including general psychiatry, developmental psychology, and others. With the advent of the child guidance movement came a strong public health perspective to childhood mental health [8]. By the mid-twentieth century, studies on psychosis in childhood, autism, manic-depressive and sleep disorders as well as various iterations of ICD and DSM brought clearer diagnostic categories, occasionally with developmental perspectives [5–7]. More systematic epidemiological studies emerging since the 1960s have mapped the prevalence of mental and behavioral disorders in children as well as paving the way for investigations into neurobiology, genetics and social determinants [6, 7, 9].

When compared to the impact of other paediatric medical disorders, the growing understanding about child and adolescent mental disorders has brought however little attention and investment from decision-makers, with health service systems generally focusing elsewhere [10, 11]. One consequence of the lack of sufficient attention and investment is that the prevalence of child and adolescent mental disorders shows no signs of diminishing; indeed, there is evidence for increasing levels of autism spectrum, depressive and substance use disorders [12–14]. While the greatest disability is in the individual child or adolescent, the adverse effects of early life mental disorders extend to their families, schools, and communities with social disruption, limited productivity, increased healthcare costs, and the diminished wellbeing in future generations [4, 7, 10].

This increasing prevalence of youth mental disorders has not been accompanied by an even remotely proportionate expansion in child and adolescent mental health services. In part, this is the result of a dramatic failure to develop an adequate child and adolescent psychiatry workforce. Worldwide, there are woefully few child and adolescent psychiatrists; in high-income countries the number of child psychiatrists is 1.19 per 100.000 youth, but in low-and middle-income countries (LMICs), where the preponderance of the world’s children and adolescents live, the number is less than 0.1 per 100.000 population [15].

Child and adolescent psychiatry is in a unique position to respond to the growing public health challenges associated with mental disorders arising early in life. However, to meet these challenges, the field must consider some changes. In this context, the future of child and adolescent psychiatry was considered by the Section on Child and Adolescent Psychiatry of the World Psychiatric Association (WPA CAP), the International Association for Child and Adolescent Psychiatry and Allied Professions (IACAPAP), the World Association for Infant Mental Health (WAIMH), the International Society for Adolescent Psychiatry and Psychology (ISAPP), the UN Special Rapporteur on the Right to Health, representatives of the WHO Department of Mental Health and Substance Abuse and other experts. We take this opportunity to outline four consensus priorities for child and adolescent psychiatry over the next decade:

1. Increase the workforce necessary for providing care for children, adolescents and families facing mental disorders.
2. Reorienting child and adolescent mental health services to be more responsive to broader public health needs.
3. Increasing research and research training while also integrating new research finding promptly and efficiently into clinical practice and research training.
4. Increasing efforts in advocacy.

### Increase the workforce

A shortage of child and adolescent psychiatrists affects all countries [15]. Even in the USA, where a national society of child and adolescent psychiatrists (AACAP) was founded 65 years ago, has less than one-fourth (currently 9000) of the number of child and adolescent psychiatrists necessary to address estimated national needs [16]. There are even fewer child and adolescent psychiatrists (less than 0.1 per 100.000 population) in LMICs [15]. There are many reasons for this situation, including: lack of training opportunities; inadequate financial compensation (child and adolescent psychiatrists earn less than other medical doctors); the time necessary for training (post graduate programs in child and adolescent psychiatry last up to 6 years after medical school); the low professional/social status of child and adolescent psychiatrists; and, stigma about mental illness as reflected by a common public perception that psychiatrists are not “real doctors” or child and adolescent psychiatric disorders are not “real illnesses” [17–19].

Although psychiatrists have historically been the mainstay of child and adolescent mental health services, there has been a welcome growth in multi-disciplinary services. In order to further extend the size and scope of the workforce of professionals committed to working with this population, more training must be available not only for child and adolescent psychiatrists, but also clinical psychologists, pediatricians, social workers, general psychiatrists, nurses, primary care practitioners, and other healthcare professionals. This expansion will be far from straightforward. There is a clear gap in available curricula adapted for multiple specialties and directed at both pre-service and in-service education for: child and adolescent psychiatrists, general psychiatrists, pediatricians, primary care and other specialty physicians, nurses, social workers, and other healthcare professionals. While manuals for *general* mental health training of non-specialists may already exist, such as the mhGAP Intervention Guide (IG) [20], there is a need for a *child and adolescent* mental health training manual (i.e. Child mhGAP-IG) adapted for multiple specialties and directed at both pre-service and in-service education. The current version of the mhGAP Intervention Guide has one module for child and adolescent mental and behavioral disorders [20], but additional materials are necessary.

More recently, there are several promising models for the integration of mental health services into primary care settings (including collaborative care models such as project ECHO [Extension for Community Healthcare Outcomes] that emphasize patient-based/real-time education (via team meetings, phone and video-teleconferenced consultations, and other preceptorships) in order to enhance the mental health competencies of primary care providers [21, 22]. These models may be useful in other settings in order to promote collaboration and mutual education among different professionals who interact with children and families.

Increasing the size of the child and adolescent mental health workforce will inevitably need other strategies, including making mental health care of children and adolescents a more attractive option for both undergraduate and postgraduate trainees, ensuring the expansion of training positions, and providing financial remuneration for child and adolescent mental health professionals that reaches levels similar to those in other areas of health care. Training programs will increasingly need to equip the child and adolescent psychiatrist of the future with a different set of skills, including a greater awareness of rapid developments in neuroscience, psychology and the social sciences as well as the necessity of adopting a greater public health perspective and extension of the work beyond the clinic setting.

### Reorienting child and adolescent mental health services

In many countries, Child and Adolescent Mental Health Services (CAMHS) are struggling to deal with growing demands and diminishing resources [15, 23, 24]. As a result, CAMHS are increasingly forced to only care for the most acutely ill individuals with mental disorders and are left with few or no resources for prevention or early intervention [25].

The main challenge for CAMHS is shortage of resources (including an acute shortage of child and adolescent psychiatrists) [15]. As demands for services are unlikely to decrease, there will be a need for CAMHS to optimize existing resources and find innovative ways to attract more resources by reengaging with public health and primary care while also addressing stigma and other challenges.

Optimizing the use of existing resources is a first step. Direct services provided by child and adolescent psychiatrists and doctoral level psychologists, are costlier than those provided by some other professionals. Therefore, the judicious balancing of service providers to include allied professionals may create the opportunity to expand services while utilizing the same limited resources. This effort must include primary healthcare providers (pediatricians, general practitioners, advanced practice nurses, and others), as well as teachers and other helping professionals. With proper preparation and training, allied professionals can provide some of the essential elements of care for the children, adolescents and families facing common mental disorders. Child and adolescent psychiatrists can then focus on: (1) initial diagnostic assessments; (2) care of the most complicated cases; and (3) support for allied professionals and their work. This strategy allows for more specialists to see the more critical and complex cases and for non-specialists to be educated on how to provide treatment and when to consult with the specialist.

Financing public health and prevention approaches to mental health are often been viewed as diverting resources from direct services for individuals already diagnosed with mental illnesses [26]. Unlike preventive interventions in other medical specialties (e.g., vaccines, anti-lipemic agents), preventive interventions in child and adolescent mental health are often felt to have minimal or only short term impacts, whereas, in reality, they have substantial long term value in obviating the need for future intensive and expensive services (e.g., inpatient and residential) [26]. In other words, fostering healthy child and adolescent development, supporting parenting, and providing early and preventive interventions will reduce the burden of child and adolescent psychiatric disorders and the attendant need for CAMHS.

Child and adolescent psychiatrists would ideally be active members of multidisciplinary public mental health teams and provide a biopsychosocial perspective on the prevention of mental health disorders and promotion of mental health. For example, child and adolescent psychiatrists commonly collaborate with schools in implementing mental health literacy programs, promoting resilience and helping children and adolescents acquire the elements necessary for healthy development and, ultimately, happy and productive adult lives.

CAMHS should not only reengage with public mental health, but also take an advantage of digital health interventions (DHI) to increase access to services. The development of DHI has been driven by three assumptions: youth prefer digital to face-to-face intervention; DHI can greatly improve access to evidence-based therapies, which may otherwise be unavailable; and, DHI appear to be more efficient and economical than center-based care. An increasing body of evidence supports the use of computers and the internet in the provision of interventions for depression and anxiety in children and adolescents [27]. Comprehensive evaluations of the effectiveness and cost-effectiveness of multiple delivery systems to address anxiety, depression, and other disorders are needed in order to shape and disseminate new approaches to DHI.

Attracting additional resources to support children and adolescents with mental disorders will require strong policy and, therefore, political support. There are examples of effective advocacy in countries where parents insist on specialized services for children with autism spectrum disorder, increase public awareness, and place societal and political pressure on decision makers [28]. These experiences should be carefully studied, as they serve as models for attracting support for other child and adolescent mental health services.

Stigma, rather than just economic considerations, may be the more persistent and pernicious cause of CAMHS resource limits. Stigma limits the allocation of resources and discourages youth and families from seeking treatment even when it is available. Stigma is often associated with misunderstandings about psychiatric illness in youth. It may also lead to the shortage of culturally-adapted, developmentally appropriate, evidence-based interventions [29]. Added to stigma are other barriers to access, engagement, early recognition and treatment, which are even more pronounced for vulnerable groups such as refugee children, street children, homeless families, youth in care programs, young offenders, gender non-conforming youth, victims of war and violence, and those facing social and economic disadvantage [30]. The complex needs of these youth highlight the importance of service coordination, joint care pathways, integrated psychosocial care, and embedding of psychiatric services within general medical services. The voices of these children and adolescents, as well as their parents, must be heard and must play a central role in shaping service planning, development, research, and evaluation.

### Integrating new perspectives into research and research training

In the last decade, there has been a great increase in research and conceptual understandings of the effects of environment, and developmental processes on brain, behavioral, emotional, and cognitive development, as well as perturbations in such development.

In the coming years, child and adolescent psychiatry will see substantial benefit from broad areas of research that have great promise for translating science into practice. Relevant areas include: genetics, developmental neuroscience, developmental psychology, epidemiology, phenotyping, new treatment targets, health economics and public mental health. Investment in these areas will facilitate prevention, early and more accurate diagnosis, and more effective and cost-effective treatment of mental disorders in children and adolescents. We examine a few examples bellow:

#### Epidemiology

Large, representative population and registry studies are providing accurate prevalence data, which indicate that there are significantly greater numbers of individuals affected by developmental psychopathology. However, more studies are necessary to offer insights into the breadth and variation in the phenotypes of childhood onset psychiatric disorders. These data will bring changes in our understanding of pathophysiology, diagnosis, and treatment. Furthermore, longitudinal studies will be necessary to provide clearer pictures of normal development and its variations in the face of developmental psychopathology. With low-and middle-income countries (LMICs) having the highest numbers of children overall and the highest numbers of children who are exposed to adverse childhood experiences [1], there is an urgent need for a better understanding of child and adolescent mental health disorders in these countries. The most sophisticated child and adolescent psychiatry research has been conducted in high-income settings, while LMICs mental health intervention studies predominantly focus on pharmaceutical trials that often take advantage of areas with little regulation [31]. The capacity to undertake child and adolescent mental health research in LMICs is improving but remains limited [32]. In order to minimize the disparity between knowledge emanating from high-resource settings and LMICs, high income groups will have to support research in LMICs to develop better surveys, cohorts, clinical trials, and cost- effectiveness studies in child and adolescent mental health.

#### Toward better phenotypes and diagnostic systems

DSM 5 and ICD 11 provide further evidence that categorical diagnosis, while robust and important, also has distinct limits [33]. The use of a categorical approach may lead to a systematic underappreciation of the importance of variations in overt symptoms and in underlying mechanisms from individual to individual. As the field tries to more fully describe the dimensions of all aspects of developmental psychopathology, the development of new models and tools for phenotyping will be necessary. Further studies will be necessary to validate these tools and translate them for use as a part of standard clinical practice. Studies using evolving brain imaging technology (e.g., fMRI, MEG, fNIR, and EEG) will provide insights into the systems biology of the brain in health and disease and will create new opportunities for defining functional elements in the brain and their role in developmental psychopathology. Further studies of the genetics (including studies on coding and non-coding regions and on epigenetics and gene expression) of psychopathology will be necessary to elucidate the etiologic understanding of disorders and phenotypes. Of note is growing evidence for the impact of stress and inflammatory processes on the developing brain and emergence of developmental psychopathology, both directly and through an impact on glial and other brain functions.

#### Therapeutics

For some time, there have been few new targets for pharmacologic interventions. This paucity of new targets is likely to change with the growing interest in the cannabinoid, glutamate and other messaging systems in the brain. These new targets will be among those identified, as inflammatory, metabolomics and genetics studies are developed and in progress. New findings may open the way for new technologies, such as optogenetics and Clustered Regularly Interspaced Short Palindromic Repeats (CRISPR)-CAS9, to create completely new strategies for treating developmental psychopathology. Environmental interventions will also continue to offer opportunities for further exploration and perhaps lead to novel strategies for the mitigation of toxic exposures (biological and psychological). It will be equally important to further develop evidence-based psychotherapies (individual and group), as well as behavioral therapies and parent training, which are directed at specific symptoms, disorders and developmental stages.

#### Health economics

Health economics will be essential justifying new investments in child and adolescent mental health services. It will require a broader perspective of the economic evaluation of interventions used in CAMHS and will need to account for costs and savings related to all societal sectors, including such as health, social, educational, and criminal justice services; and other impacts such as loss of productivity, family instability, and lack of self-sufficiency. Better integration of economic evaluations into clinical trials using generic outcome indices, such as QALYs (quality-adjusted life years using for example the CHU9D or Child Health Utility instrument) will be particularly helpful in making the case for allocating resources for CAMHS.

#### Research in prevention

Since the majority of lifetime mental illnesses develop before adulthood, effective prevention targeted at children and adolescents is likely to generate greater personal, social and economic benefits than interventions at any other time in the life course. Prevention research can explore and provide evidence for a broad range of potential preventive strategies (e.g., school based, family, social system etc.) in different cultures and regions. Careful planning will allow for the evaluation of safety, efficacy and cost effectiveness in standard trials. A developmental perspective should be a key underpinning of prevention research, providing insights into the pathways, continuities, and changes in normal and pathological processes over the life span [34]. It will move research away from the notion of a single causal agent and will attempt to examine different and sometimes interacting causal factors as well as identify optimal points for intervention. Given this complexity, it is expected that child and adolescent psychiatry and multiple other disciplines will work together to succeed in comprehensive preventive research trials.

### Greater leadership in advocacy

Development and implementation of a multi-sector policy and strategic action plans for child and adolescent mental health is a high priority. In this process, the role of child and adolescent psychiatrists must be clearly defined. Multi-sector mental health policy is best characterized by a holistic, evidence-based approach to the identification and treatment of mental disorders, with specific attention to prevention, early intervention, and rehabilitation for psychiatric disorders [35]. To be effective, it is important that a multi-sector child and adolescent mental health policy be reflected in all levels of the government and community, and include: human rights, service organization and delivery, development of human resources, sustainable financing, civil society and advocacy, quality improvement, information systems, program evaluation and plans to address stigma. Political will and commitment from policy makers, community agencies, NGO’s, the government and other sectors will be necessary in order to arrive at a shared policy framework for concrete policies and actions.

Child and adolescent psychiatrists can and should play a greater leadership role in advocating for human rights. The United Nations Convention on the Rights of the Child is at the core of the transnational commitment to protecting children and adolescents [36]. It guarantees children the full range of human rights and sets international standards for the rights of the individual child. Advocacy around the prevention of psychological trauma is a particularly important focus given that early childhood exposure is likely to affect formative developmental processes in a manner that impairs the foundation of future growth and that may have intergenerational consequences. Institutional care for children during the first 5 years of life represents a special risk that should be eliminated with investments in community-based services for families at risk, including for families living in poverty and those with young children facing developmental and other disabilities [37].

Early childhood interventions (including those addressing mental health and socio-emotional development) should be integrated into the systems for general healthcare with adequate funding; they can and should be provided as a core element of the larger investment in the health, economic prosperity, and safety of each nation and community. The infant, by reason of his/her physical and mental immaturity and absolute dependence, needs special safeguards and care, including appropriate legal protection [31]. Caregiving relationships that are sensitive and responsive to infant needs are critical to human development and thereby constitute a basic right of infancy. Sound and supported parenting is a critical part of safe and effective childrearing and must be a central theme in the developmental model offered by child and adolescent psychiatry.

Adolescents should be recognized as representing a special population. On the one hand, the community must respect their developmental rights and movement toward full autonomy; on the other, there must be a recognition that their capacities may be limited in some functional areas. Adolescents therefore need a different approach in fostering healthy development and resilience. They should be protected from violence and exploitation, but approaches must take into account their emerging competencies and capacities developing during this period of life. In many countries, mental health services for adolescents either do not exist or constitute low quality residential and in-patient services, sometimes violating human rights and relying solely on pharmacologic therapies [38]. Such services do not represent the current knowledge and acceptable standards for treatment. All evidence suggests that appropriate care can and should be offered through community-based services that are respectful of adolescents and attentive to their evolving capacities and autonomy, as well as their rapidly changing physical, emotional, behavioral, social, academic/vocational, and sexual functioning [38]. Adolescent mental health services should ensure respect for adolescents’ rights to privacy and confidentiality, address their different cultural needs and expectations, and comply with ethical standards.

## Conclusions

Although child and adolescent mental disorders are common and effective treatments are now available, services for those in need are largely unavailable. The failure to address the mental health needs of children and adolescents represents a failure to address a substantial public health problem and constitutes a profound and broad-based failure to meet intrinsic societal responsibilities. Child and adolescent psychiatry, as a medical specialty with a strong neurobiological, psychosocial and developmental framework, is in a unique position to bring about change. Child and adolescent psychiatry is well-suited and well-prepared to take up the leadership role in this time of transition. This role will be enhanced by expanding the number of child and adolescent psychiatrists, as well as building a broader child and adolescent mental health workforce, an engagement with broader health service systems, a greater emphasis on preventive approaches, adapting new research into practice and taking on greater leadership in advocacy. It will require child and adolescent psychiatrists to work differently with disciplines outside of psychiatry, including other physicians and colleagues in related mental health disciplines. Together, we can work more effectively to bring social and political attention, as well as investment at local, national and global levels to assure proper care of child and adolescent mental disorders.

By taking a leadership role in child and adolescent mental health and beyond, child and adolescent psychiatry will enhance healthy and productive development of our children and adolescents and the entire world community.

## Abbreviations

WPA CAP: Child and Adolescent Psychiatry of the World Psychiatric Association
IACAPAP: International Association for Child and Adolescent Psychiatry and Allied Professions
WAIMH: the World Association for Infant Mental Health
ISAPP: International Society for Adolescent Psychiatry and Psychology
UN: United Nations
WHO: World Health Organization
DALYs: disability-adjusted life years
ICD: International Classification of Diseases
DSM: Diagnostic and Statistical Manual of Mental Disorders
LMIC: low-and middle-income countries (s)
CHO: Extension for Community Healthcare Outcomes
CAMHS: Child and Adolescent Mental Health Services
DHI: digital health interventions
fMR: Ifunctional magnetic resonance imaging
MEG and EEG: magneto- and electroencephalography
NGO: non-governmental organization
